# Induced Pluripotent Stem Cell (iPSC)-Based Neurodegenerative Disease Models for Phenotype Recapitulation and Drug Screening

**DOI:** 10.3390/molecules25082000

**Published:** 2020-04-24

**Authors:** Chia-Yu Chang, Hsiao-Chien Ting, Ching-Ann Liu, Hong-Lin Su, Tzyy-Wen Chiou, Shinn-Zong Lin, Horng-Jyh Harn, Tsung-Jung Ho

**Affiliations:** 1 Bioinnovation Center, Buddhist Tzu Chi Medical Foundation, Hualien 970, Taiwan; scata0726@hotmail.com (C.-Y.C.); sharkzoe@yahoo.com.tw (H.-C.T.); sagianne@gmail.com (C.-A.L.); suhonglin@gmail.com (H.-L.S.); twchiou@gms.ndhu.edu.tw (T.-W.C.); shinnzong@yahoo.com.tw (S.-Z.L.); 2 Department of Medical Research, Hualien Tzu Chi Hospital, Hualien 970, Taiwan; 3 Neuroscience Center, Hualien Tzu Chi Hospital, Hualien 970, Taiwan; 4 Department of Life Sciences, National Chung Hsing University, Taichung 402, Taiwan; 5 Department of Life Science, National Dong Hwa University, Hualien 974, Taiwan; 6 Department of Neurosurgery, Hualien Tzu Chi Hospital, Hualien 970, Taiwan; 7 Department of Pathology, Hualien Tzu Chi Hospital and Tzu Chi University, Hualien 970, Taiwan; 8 Department of Chinese Medicine, Hualien Tzu Chi Hospital, Hualien 970, Taiwan; 9 Integration Center of Traditional Chinese and Modern Medicine, Hualien Tzu Chi Hospital, Hualien 970, Taiwan; 10 School of Post-Baccalaureate Chinese Medicine, Tzu Chi University, Hualien 970, Taiwan

**Keywords:** iPSC, neurodegenerative diseases, drug screening

## Abstract

Neurodegenerative diseases represent a significant unmet medical need in our aging society. There are no effective treatments for most of these diseases, and we know comparatively little regarding pathogenic mechanisms. Among the challenges faced by those involved in developing therapeutic drugs for neurodegenerative diseases, the syndromes are often complex, and small animal models do not fully recapitulate the unique features of the human nervous system. Human induced pluripotent stem cells (iPSCs) are a novel technology that ideally would permit us to generate neuronal cells from individual patients, thereby eliminating the problem of species-specificity inherent when using animal models. Specific phenotypes of iPSC-derived cells may permit researchers to identify sub-types and to distinguish among unique clusters and groups. Recently, iPSCs were used for drug screening and testing for neurologic disorders including Alzheimer’s disease (AD), amyotrophic lateral sclerosis (ALS), spinocerebellar atrophy (SCA), and Zika virus infection. However, there remain many challenges still ahead, including how one might effectively recapitulate sporadic disease phenotypes and the selection of ideal phenotypes and for large-scale drug screening. Fortunately, quite a few novel strategies have been developed that might be combined with an iPSC-based model to solve these challenges, including organoid technology, single-cell RNA sequencing, genome editing, and deep learning artificial intelligence. Here, we will review current applications and potential future directions for iPSC-based neurodegenerative disease models for critical drug screening.

## 1. Neurodegenerative Diseases: the Unmet Medical Need

Neurological disorders represent a substantial unmet medical need, notably as the population ages worldwide. Many neurodegenerative diseases, including Alzheimer’s disease (AD), Parkinson’s disease (PD), Huntington’s disease (HD), spinocerebellar atrophy (SCA), spinal muscular atrophy (SMA), and amyotrophic lateral sclerosis (ALS) lack any significant or truly effective treatments.

Genetically modified human neuronal cell lines and primary animal neuronal cells are typically the first target for drug screening; animal models are typically used for documenting pre-clinical efficacy. The pathogenic mechanisms underlying neurodegenerative diseases are complex and still largely unknown. Most human cell lines and animal models were established with artificial methods and/or genetic overexpression strategies that may not fully represent human disease pathology. Injected or overexpressed amyloid peptides may elicit a substantial inflammatory response in a mammalian brain, but this finding may or may not have relevance to the mechanisms underlying AD. Furthermore, the human central nervous system is substantially different from those of standard laboratory animals, and primate testing is of course prohibitive. As such, novel disease models with iPSCs derived from human somatic tissues may be able to recapitulate disease phenotypes and mechanisms to facilitate drug development.

## 2. iPSC-Derived Neurons and Glia as in Vitro Models of Neurodegenerative Diseases

Human iPSCs were first established in 2007 in the laboratory of Professor Shinya Yamanaka [1,2]. Overall, iPSCs are similar to embryonic stem cells (ESCs) [3], as they are pluripotent and are capable of self-renewal in vitro. Notably, various types of somatic cells such as skin fibroblast and peripheral blood mononuclear cells can be induced to revert back to an ESC-like state by the delivery of specific reprogramming genes [4,5,6]. As such, iPSC technology can address issues related to the shortage of and complex social issues involved with the use of ESCs, which can only be obtained from abandoned embryos that were to be used for in vitro fertilization.

**Figure 1:**
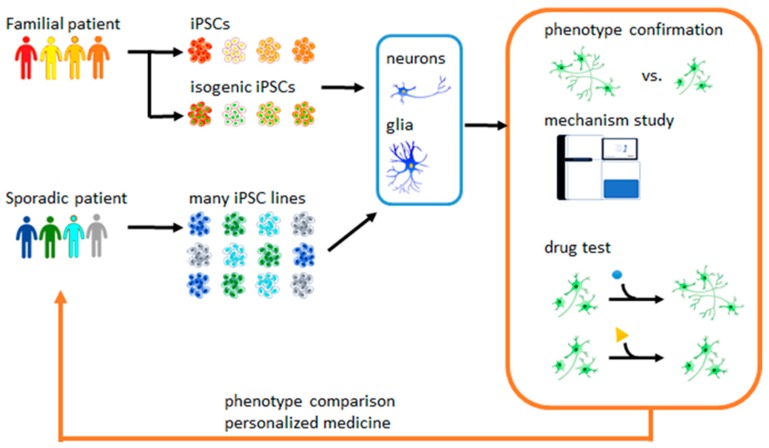
Apply induced pluripotent stem cells (iPSC)-derived neurons/glia for neurological disease phenotype confirmation, mechanism study, and drug test.

Within 10 years, iPSCs have been featured in developmental studies, cell transplantation trials, and disease modeling. Among the complex and varied conditions addressed by iPSC technology, they have been used for transplantation to treat macular degeneration, corneal transplants, PD, heart failure, diabetes, and immunotherapy [7]. For in vitro disease modeling, patient-derived iPSCs are in wide use for the study of all aspects of cell biology of disease, phenotype studies, analysis of genetic function, and ongoing drug screening (Figure 1) [8]. Previous neurodegenerative disease models, including transgenic animals and immortal neuronal cells that overexpressed human cytopathic mutated proteins, might have identified disease mechanisms that differed from those in patients. Using patients’ somatic cell-derived iPSCs, researchers can obtain pathogenic cell types directly from patients that may potentially share the same disease mechanisms for drug discovery.

For neurodegenerative disease modeling, iPSCs were sequentially differentiated into various kinds of neurons and glia with an effort made to mimic the process of central nervous system (CNS) development. iPSC-derived differentiated neurons and glia were evaluated for their capacity to model several challenging neurodegenerative diseases, including AD, HD, PD, ALS, SCA, and SMA, among others (briefly summarized in Table 1) [8].

## 3. iPSCs Can Differentiate into Neurons and Glia to Model Central and Peripheral Nervous Systems

For neurodegenerative disease modeling, iPSCs were differentiated into pathogenic cell types in an attempt to recapitulate disease phenotype. To promote differentiation of iPSCs into CNS neurons and glia, the expression of activin/nodal and bone morphogenetic protein (BMP) are inhibited; this will prohibit differentiation into mesoderm or endoderm and promote the generation of neuroectoderm tissue. The most widely used neural stem cell (NSC) differentiation protocol was developed by Professor Lorenz Studer, who called it “dual SMAD inhibition” (dSMADi); using this method, one can generate CNS NSCs to greater than 95% purity [9]. The fate of naïve dSMADi-derived NSC was the dorsal forebrain; then, these cells could develop into cortical neurons. To guide these NSCs into other CNS neurons to their final destination, an additional step called “patterning” is applied. Basically, the CNS neural tube development takes place along a dorsal-ventral (D-V) and anterior-posterior (A-P) axis; the D-V axis is controlled by signals from wingless-int (Wnt), BMP, and sonic hedgehog (Shh) factors; the A-P axis is controlled by Wnt and retinoic acid signaling pathways. Dosed combinations of these morphogens provide patterning information and promote the generation of ganglionic eminence neurons, serotonin neurons, dopaminergic neurons, Purkinje cells, and motor neurons [10,11,12,13,14,15,16,17,18,19,20,21,22,23,24,25,26,27,28,29,30,31,32,33,34]. For region-specific glial cell differentiation, patterned NSCs should be maintained for several months and ultimately switched from neurogenesis to gliogenesis [35,36,37,38,39,40,41,42,43,44,45]; high levels of nuclear factor 1A (*NF1A*) undergo a decline during this transition [46,47,48].

For generating cells for the peripheral nervous system (PNS), the iPSCs are induced to generate neural crest cells (NCCs), which can then develop into peripheral neurons, glia, and non-neural cell types. In response to the low-level activation of Wnt and BMP and the inhibition of activin/nodal, Tchieu and colleagues reported that more than 60% of the iPSC-derived NCCs [49,50] ultimately differentiate into enteric neurons, sensory neurons, and Schwann cells, which are useful for disease modeling [51,52,53,54,55,56,57,58].

## 4. Patient iPSCs to Modeling and Drug Screening for AD

At the time of writing, approximately 5% of those over 65 years of age suffer from dementia and 50–60% of those with dementia will ultimately be diagnosed with AD. Currently, there are only two approved clinical treatments for AD, acetyl-cholinesterase inhibitors and the *N*-Methyl-*D*-aspartate receptor antagonist; both have very limited therapeutic effects. As an effort toward developing novel therapeutic compounds, iPSCs from patients with AD or Down Syndrome (DS) were induced to differentiate into cortical neurons; this resulted in a recapitulation of characteristic AD phenotypes, including amyloid-β (Aβ) aggregates and neurofibrillary tangles (NFTs). Likewise, iPSC-derived neurons from patients with DS exhibited accelerated AD-related pathologies, including the Aβ aggregates and dysregulation of Tau protein; degenerating synaptic functions and stress vulnerabilities were also observed in these neurons [59,60,61]. These findings suggested that iPSC-derived neurons from patients with DS neurons may serve as an important model for screening novel compounds for the capacity to inhibit Aβ aggregates and Tau dysregulation. In our previous work, we demonstrated that “*n*-butylidenephthalide”, a major compound from the neuroprotective Chinese herb *Angelica sinensis*, could rescue DS induced pluripotent stem cells (iPSC)-derived neurons from Tau-related pathology and enhance canonical Wnt pathway activation [59]. Furthermore, iPSC-derived forebrain neurons from *APP* and *PSEN1* mutated AD patients were evaluated in phenotype studies and revealed a higher Aβ42/40 ratio and Aβ oligomer accumulations [62,63,64,65,66,67,68,69]. Increased Tau and hyper-phosphorylated Tau protein levels, early endosome accumulation, GSK3β overactivation, and increased reactive oxygen species (ROS) production were also observed in amyloid precursor protein (APP)-mutated forebrain neurons [62,63,65,66,70]. AD-associated astrocytes were themselves neurotoxic and capable of damaging healthy neurons by reducing lactate secretion, increasing Aβ release and cytokine production, and inducing abnormal calcium flux [63,71,72]. Although there are many publications that suggest that iPSC-derived neurons from AD patients have reproduced disease-associated phenotypes, the complexity and poor characterization of AD itself limits the use of iPSCs in in vitro models for drug screening. In 2013, Kondo and colleagues demonstrated that iPSC-derived neurons from familial versus sporadic AD cases exhibit different cytopathic phenotypes and drug responsiveness. Furthermore, the neurons displayed different levels of cellular stress and exhibited different responses to docosahexaenoic acid (DHA), an omega-3 unsaturated fatty acid with protective effects against ROS, depending on the degree of Aβ oligomer accumulation. Specifically, DHA rescued the APP-E693ΔAD neurons from damage to reactive oxygen species (ROS), synaptic degeneration, and cell death [63]. These results suggest that AD may have multiple sub-types with clinical different responses. With this understanding, we might keep in mind that several agents that failed in global clinical trials may still have potential to treat some AD sub-types. Toward that end, iPSC-based models have the potential to become personal precision models which may recapitulate early processes related to AD process and ultimately assist with the identification of suitable treatment strategies. In 2017, this group of researchers used 13 iPSC-derived lines from AD patients for drug screening. In response to the transient expression of neurogenin 2 (NGN2), neurons were generated within approximately one week. A library that included 1258 pharmaceutical compounds was applied to the iPSC-derived AD neurons with outputs including Aβ40 and Aβ42 secretion and the Aβ42/40 ratio. After two rounds of screening, 27 potential therapeutic compounds were grouped into 10 clusters; 6 lead compounds were chosen due to their capacity to reduce Aβ40 and Aβ42 levels in most of the 13 sets of AD neurons. Finally, the cocktail containing bromocriptine (a dopamine receptor activator), cromolyn (a compound for preventing eye allergy), and topiramate (a clinical compound for epilepsy therapy) was revealed to reduce the Aβ42/40 ratio. Unfortunately, the cocktail of three lead compounds had the capacity to reduce the Aβ42/40 ratio in iPSC-derived neurons from patients with familial AD and not those from patients with sporadic AD [64]. Furthermore, in recent years, the role of the Aβ aggregates as the major therapeutic target of AD has been questioned. Therefore, the definition of appropriate AD phenotypic markers has become a major goal of drug screening procedures.

## 5. iPSCs for Modeling and Molecular Mechanism Studies for PD

Approximately 1–2% of those 60 years and older may carry a diagnosis of PD, which is a CNS neurodegenerative disorder that results in the loss of motor function. More than one-third of current PD patients may also be diagnosed with dementia, depression, anxiety, and other emotional symptoms that as a group are called Parkinsonism. The loss of dopaminergic neurons (DA-neurons) in the midbrain is the major cause of motor symptoms in PD. Levodopa and dopamine agonists are provided to supply dopamine and delay degeneration secondary to PD; at later stages, deep brain stimulation results in prolonged motor functions and improved quality of life for PD patients. However, there is still no treatment that delays DA neuron death; the cause of this disease is also largely unknown. To explore the mechanisms underlying PD and PD-related dysfunction, PD-associated iPSCs were differentiated into DA neurons for disease modeling. For familial PD, iPSC-derived DA-neurons with mutations in *SNCA*, *LRRK2*, *PINK1*, and *Parkin* were characterized by cytosolic phenotypes [73,74,75,76,77]. *LRRK2-*mutated DA-neurons displayed mitochondrial DNA damage and dysfunction, DA neuron degeneration, and deficient autophagy [73,76,77]. *PINK1* and *Parkin* mutations might cause neurite degeneration, mitochondrial enlargement with inclusions, and DNA damage and dysfunction in DA neurons [73,75]. *SNCA* mutations result in α-synuclein protein accumulation [74]. Some DA neurons were excessively vulnerable to cell stress in long-term in vitro culture. Astrocytes derived from *LRRK2* G2019S progenitors exhibited autophagy dysregulation, as α-synuclein accumulation and secretion phenotypes result in neuronal toxicity [78]. Coenzyme Q10, rapamycin (mTOR inhibitor), and GW5074 (cRaf1 kinase inhibitor) could rescue *PINK1* and *LRRK2* DA neurons from mitochondrial dysfunction [73]. Similar phenotypes were also observed in some sporadic cases. However, there were still some sporadic PD iPSC-derived DA neurons did not exhibit these cytopathic phenotypes. Environmental factors and aging may be associated with the PD phenotype that are not recapitulated in the iPSC-based models. Neurons derived from iPSCs overexpressing progerin, a truncated version of lamin A that contributes to the underlying pathology of the premature aging disease Hutchinson–Gilford progeria syndrome (HGPS), exhibited accelerated aging, appearing suitable for specific phenotype recapitulation [75]. Of note, the HGPS patients did not exhibit early neuronal aging phenotypes, and as such, the similarity between natural and progerin-induced neural aging is still unclear. Furthermore, the progerin-induced aging DA neurons did not express classical PD cytopathies such as α-synuclein accumulation. Until now, the PD specific cytopathies are observed only in iPSC-derived neurons from patients with familial PD and few sporadic ones.

## 6. iPSCs for Screening of Therapeutic Compounds for ALS

ALS is a relatively rare disease associated with the progressive loss of motor function; fewer than 20% ALS patients survive for 5 years after diagnosis. Five to 10% of the cases of ALS are familial, and more than 90% of ALS cases are sporadic with unclear etiology. iPSC-derived motor neurons from patients with sporadic ALS carrying mutations in genes including *SOD1*, *TDP-43*, *FUS*, and *c9orf72* were used for drug screening and mechanistic studies [79,80,81,82,83,84,85,86,87,88,89,90,91]. The observed neuron-specific cytopathies included nerve fiber dysregulation and neurite degeneration [81,88,91,92]. Misfolded protein aggregates were detected in iPSC-derived motor neurons with mutations in *TDP-43*, *SOD1*, and *FUS* [80,81,82,83,91]. *FUS* mutation also caused axonal transport defects and stress granule formation [82]. Astrocytes and oligodendrocytes derived from iPSCs from patients with ALS were also highly neurotoxic, promoting motor neuron death [86,93,94]. Tyzack and colleagues found that the *SOD1* mutation disrupted the EphB1 pathway, which can mediate anti-inflammation response for neural protection, in astrocytes [95]. Anacardic acid and HDAC6 inhibitors exerted protective effects and rescued *TDP-43* and *FUS* motor neurons from phenotypes [82]. Two strategies were recently described by Imamura and Fujimori for novel drug screening. Specifically, the group established 12 ALS-derived iPSCs that were differentiated into motor neurons within 7 days using a gene overexpression method. After screening 1416 compounds, they identified 7 compounds that target the Src/c-Abl pathway and have the capacity to improve motor neuron survival [92]. Fujimori and colleagues established more than 40 ALS iPSC lines. An automatic imaging process was applied to identify motor neuron pathology and showed that iPSCs from ALS clustered into several unique phenotypes. Four major markers of neuropathology (neurite regression, protein aggregates, stress granules, and cytotoxicity) were measured in response to 1232 compounds, and 9 compounds were identified as resulting in improvements in *FUS-* and *TDP-43*-mutant motor neurons. Phenotypes of 32 sporadic ALS iPSC-derived motor neurons were measured and clustered into *FUS*, *TDP-43*, and *SOD1* mutated-like groups and then screened for responses to various compounds. The repurposed drug, ropinirole (a dopamine receptor activator), was identified in this study and has been in clinical trials for ALS since 2018 [88]. Neuromuscular junction (NMJ) degeneration is the early cytopathy of ALS that directly influences motor function. Reports also suggested that cytopathic cells, motor neurons, and skeletal muscles in the NMJ influenced each other to promote the progression of ALS [96,97]. Thus, NMJ models represent in vitro tools for clarifying the early progression of ALS. For NMJ models, iPSC-derived motor neurons and skeletal muscle were used [89,90]. Osaki and colleagues applied a chip microfluidic device, which assembled iPSC-derived skeletal muscle together with iPSC-derived motor neurons. With the optogenetic control of gene expression, neuronal firing was elicited by light, and the resulting muscle contraction response was measured as an index of neuromuscular function. Two compounds, rapamycin and bosutinib (Src/c-Abl pathway inhibitor), exhibited a therapeutic impact on NMJ responses [90].

## 7. Modeling of Rare Neurodegenerative Diseases from iPSCs

Models featuring iPSCs have been used to explore mechanisms underlying Huntington’s disease (HD) [98,99,100,101], spinocerebellar atrophy (SCA) [102,103,104], spinal muscular atrophy (SMA) [105,106,107,108], Hirschsprung disease [49], and familial dysautonomia [57]. Disease-specific phenotypes and vulnerabilities were described in iPSCs derived from patient cells in most reports. For example, Ishida and colleagues demonstrated that SCA6 Purkinje cells were associated with vulnerability to thyroid hormone depletion-specific neurite degeneration. The clinical ALS therapeutic compound riluzole and thyrotropin-releasing hormone may rescue Purkinje cells from disease phenotypes [102]. Htt aggregates, autophagy, lysosome and metabolism dysregulation, and dysregulated adhesion were observed in HD γ-Aminobutyric acid (GABA) neurons, ultimately leading to caspase activation and cell death [98,99,100,101]. Decreased survival motor neuron (SMN) protein levels, degenerated neurites, and delayed neural differentiation were found in SMA motor neurons [105,106,107,108]. SMA iPSC-derived astrocytes had an abnormal morphology, reduced neurotrophic factor secretion, and elevated calcium flux, which are suggested to lead to motor neuron damage [109]. Valproic acid, an Histone deacetylases (HDAC) inhibitor used to treat epilepsy, could protect motor neurons against SMA cytopathies [105,108]. Glial fibrillary acidic protein (GFAP) mutation resulted in protein aggregation in astrocytes from models of Alexander disease. Thus, these astrocytes secreted higher levels of inflammatory cytokines and other molecules that caused immunoreactivity and inhibited myelination [110,111].

## 8. Current and Future Challenges: Use of iPSCs for Drug Screening for Neurodegenerative Diseases

### 8.1. Challenges and Strategies to Accelerate Neural Maturation

**Figure 2:**
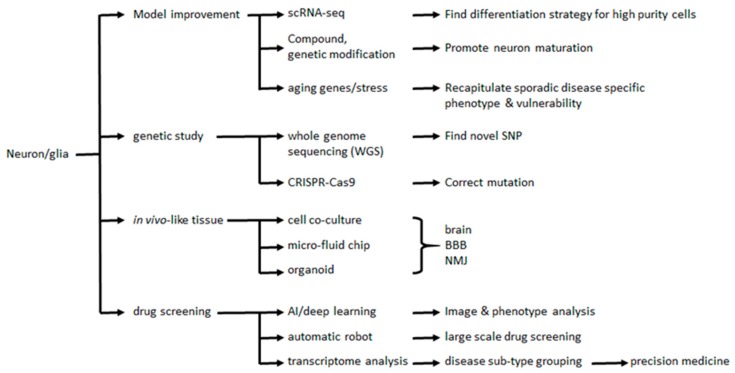
Combine novel technologies and iPSCs for disease model improvement, genetic studies, make complex neuronal organoids, and large-scale drug screening. scRNA: single cell RNA, SNP: single nucleotide polymorphism, BBB: blood–brain barrier, NMJ: neuromuscular junction.

Although the iPSC technology is a powerful tool, there are various challenges ahead (Figure 2). Among these challenges, collecting samples from patients, establishing iPSC cultures, and inducing specific neuron and glial differentiation is extremely time-consuming (>30 d) and effort-intensive, which are two points that reduce enthusiasm and feasibility for drug screening. To overcome this challenge, Notch and γ-secretase inhibitors have been shown to shorten the maturation period of most neuronal cell types [112,113]. Another approach includes the overexpression of neural specific genes; the overexpression of *Ngn2* accelerates the production of both forebrain and motor neuron cells to within 10 days [64,92]. However, the authors did not measure the gene expression patterns or overall disease cytopathies to clarify whether *Ngn2* overexpression influences the neuronal properties or disease phenotypes. Moreover, the usage of a lentivirus-mediated transgene strategy to deliver *Ngn*2 may damage the cell genome at multiple sites of chromosomes [64,92]. Thus, the accuracy of *Ngn2*-induced neuronal maturation in disease modeling remains questionable.

### 8.2. Challenges to Recapitulate Disease Phenotypes in Sporadic and Late-Onset Neurodegenerative Disease Models

Various reports have suggested that iPSC-derived neurons can reproduce the phenotypes of neurodegenerative disease. However, most of these cases were from the familial cases associated with specific genetic mutations. For sporadic neurodegenerative diseases, generating cytopathic cells with disease-related phenotypes remain a challenge. In familial cases, because of genetic mutations, the phenotypes may be dominant and serious, thereby causing early-onset disease pathology in both patients and iPSC models. Moreover, it is not difficult to speculate the potential disease phenotypic markers with familial cases according to the genetic database. However, in sporadic cases, the causes of disease may be associated with genetic risks, epigenetics, aging, and environmental factors, resulting in a late-onset phenotype that is difficult to be recapitulated in tissue culture. Furthermore, the progression of sporadic neurodegenerative diseases is extremely complex, making it difficult to identify causes and discover ideal disease markers.

### 8.3. Induce Aging in Late-Onset Neurodegenerative Disease Models to Recapitulate Phenotypes

We know that environmental factors and aging may be two major factors associated with the development of sporadic neurodegenerative diseases. However, the generation of iPSCs erases the epigenetic markers from somatic cells and reverts the cells to the “fetal” stage. iPSC-derived neurons do not exhibit aging-related features such as genomic instability, telomere degeneration, or mitochondria function decay. Therefore, strategies for accelerating aging are needed to mimic sporadic or late-onset neurological diseases. To date, the most workable strategy is the overexpressing of proteins that are associated with aging such as progerin to accelerate cellular aging [75]. However, this strategy does not fully recapitulate natural aging. For example, few early neurodegenerative phenotypes exist in the CNS neurons of patients with HGPS, and nuclear aging is not always observed in normal neuronal aging processes. However, it is still a helpful strategy for recapitulating PD-like phenotypes in sporadic iPSC-derived DA-neurons. Another potential strategy is “transdifferentiation.” Many reports suggested that the direct conversion of somatic cells (such as skin fibroblasts) into neurons (called induced neurons [iNs]) without back-processing into iPSCs permitted the retention of epigenetic markers and disease phenotypes in iN-based models [114]. However, the induction efficiency, purity, and cell numbers from iN-based strategies are not sufficient for detailed studies and drug screening.

### 8.4. Environmental Factors Promote Disease-Specific Vulnerabilities in iPSC-Based Sporadic Neurodegenerative Disease Models

The extent of the dominance of environmental factors on neurodegenerative disease remains unclear. Although environmental factors are complex and multi-aspect variables, some researchers concluded that environmental factors could finally lead to oxidative stress. Clinically, oxidative stress markers were observed in cerebrospinal fluid or blood plasma from patients with neurodegenerative diseases. To mimic environmental influences on iPSC models as much as possible, ROS, neurotoxins, the induction of overexcitation, and the depletion of medium components have all been applied, and specific vulnerabilities have been identified in various iPSC-based neurodegenerative disease models [75]. These reports suggested that environmental factors could promote the appearance of disease-specific phenotypes in iPSC-based sporadic models, making them potentially useful for drug screening.

### 8.5. Disease Subtype Grouping Provides Reliable Phenotypes for Drug Screening and Precision Medicine

For drug screening, the choice of an appropriate and early-onset major phenotype as the marker to measure the therapeutic effect of tested drugs is extremely important. Most neurological diseases display one or more associated phenotypes including protein aggregates, stress granules, apoptosis, membrane leakage, neurite degeneration, and functional loss in the late stage. Sporadic neurodegenerative diseases may also have various different associated phenotypes according to the initial causes of the diseases, similarly as familial neurodegenerative diseases. However, this is difficult to address because of our limited knowledge on neurodegenerative diseases. For example, in both DS and AD, both Tau hyperphosphorylation (the initial stage of neurofibrillary tangles) and abnormal Aβ42/40 ratio (a measure of early cytopathy) were observed, although neither of these cytopathies were identified to be correlated with late stage neuronal toxicity. To discover the relationship between early and late phenotypes, a large amount of mechanism studies should be undertaken before applying the findings to drug screening. Another potential strategy is identifying disease indices from multi-aspects/disease stages to sub-group sporadic cases into clusters for drug screening before we completely understand the disease mechanisms. Fujimori and colleagues first demonstrated that the speed of the progression of phenotypes in sporadic ALS iPSC models could be correlated with patients’ clinical disease progression to increase the reliability of iPSC models on ALS [88]. Moreover, sporadic ALS motor neurons can be classified into FUS-ALS-, TDP43-ALS-, and SOD1-ALS–like sub-groups according to the progression of the associated phenotypes. The repurposed drug ropinirole has therapeutic effects on FUS- or TDP-43-like ALS motor neurons but not SOD1-like sporadic ALS motor neurons [88]. This research suggested that associated disease markers (e.g., neurite degeneration, cytosolic stress, mitochondrial dysfunction, oxidative stress, cell leakage, protein accumulation, metabolism dysregulation, cell apoptosis, and electrophysiological function degeneration) should be discovered and fully identified to group the sporadic types of ALS into clusters. Research using clustered sporadic cases may provide reliable materials for further drug discovery and permit the development of personalized therapeutic strategies.

### 8.6. Glial Cells and Microglia Play Key Roles in Disease Progression

Another major barrier to recapitulating neurodegenerative disease phenotypes is the establishment of interactions among neurons, glial cells, and immune cells in in vitro models. Until recently, most iPSC-based neurodegenerative models were relatively primitive in nature. For example, cortical neurons or forebrain astrocytes from iPSC-derived cells from patients with AD, motor neurons derived from patient with ALS, and DA neurons associated with PD were fairly simple and were unable to mediate complex cell–cell interactions within CNS, especially for challenging cortical brain diseases such as AD, as mentioned previously. Glial cells including astrocytes and oligodendrocytes in the CNS and Schwann cells in the PNS provide several important functions such as nutrition transfer, neurite prune, waste clearance, and neurite myelination. Several studies demonstrated the neurodegenerative disease iPSC-derived CNS glial cells expressed disease-related phenotypes, as described previously. However, months are required to differentiate CNS glial cells, even with the overexpression of the novel gliogenesis gene *NF1A* [46,47,48]. This drawback limits the application of iPSC-derived glial cells for disease modeling and drug screening. Thus, discovering the novel strategy for promoting gliogenesis is extremely important for large-scale drug screening. Schwann cells are derived from NCCs instead of NSCs. A high-efficiency differentiation method for Schwann cells requires further development [50,51,52,54,55,58,115].

Microglia are the resident macrophages in the brain that regulate the CNS immune responses. Many neurodegenerative diseases were reported to be related to CNS immune dysregulation and chronic inflammation. People with metabolic problems may have more immune proteins in the plasma and thus higher accumulation of such proteins in the CNS via the blood–brain barrier (BBB), thereby causing CNS immune response and increasing the risk of neurodegenerative diseases. Differentiation protocols for generating iPSC-derived microglia were recently established. The major sources of microglia are the yolk sac, an extra-embryonic membrane, and mesoderm-derived hematopoietic stem cells. However, some issues exist concerning the characteristics of cells derived from these two sources, and the differentiation efficiency requires further improvements for application to CNS disease modeling or drug screening. Some reports applied patient macrophages for neurological disease modeling, but microglia are not yet available for this purpose [116].

### 8.7. Generation of 3D Tissue-Like Organoids for CNS Drug Screening

To construct a complex and organized CNS-like model, organoid generation, tissue engineering, 3D printing, and chip technologies should be included into the disease models. Self-organized brain organoid technology provides an integrated brain tissue-like structure containing neurons, glial cells, and sometimes microglia and other CNS cell types [117,118,119,120]. With this technology, there is no need to differentiate various cells types individually and place them together with chip or 3D printing. However, although numerous methods have been developed to improve the reproducibility of CNS organoid systems, there is still need for additional studies to improve the quality and consistency of this approach [66,119,120,121,122,123,124,125,126,127,128,129,130,131,132,133,134]. Qian and colleagues designed a small bioreactor to separate each organoid into a single chamber, which permitted a high level of quality control [135]. However, for drug screening, methods will be needed for generation of large-scale, high-quality, highly reproducible organoids. Likewise, recent publications report successful applications that combine iPSC and chip technologies to model the BBB [136], neural–glia interactions [136], and neuromuscular junction [90] models. Two or more types of iPSC-derived cells can be placed together in well-designed microfluid channels to mimic specific cell–cell interaction niches in the CNS. Most chips were sufficiently small and thin to permit the observation of the cell morphologies, and assays such as immunofluorescence, apoptosis, and neuroelectronic response analyses can be performed directly on the chip. For application to large-scale preparation, an automatic system could be introduced for medium replacement, cell seeding, and high throughput candidate compound screening [137]. For disease phenotype measurements, deep learning technologies may have the potential to use phenotype imaging and calculation in combination with chip methodologies for large-scale screening.

### 8.8. Novel Biomolecular Technologies Benefit Neurodegenerative Disease Research

For detailed disease mechanistic studies, the novel genome editing tools such as zinc finger nuclease, transcription activator-like effector nucleases, Clustered Regularly Interspaced Short Palindromic Repeat/CRISPR associated protein 9 (CRISPR/Cas9), piggybac transposon, and bacterial artificial chromosomes provide genetic tools that facilitate gene modifications. Next-generation sequencing technologies provide tools to assess the whole-genome information and transcriptome of each single cell. Single-cell transcriptome information can improve the differentiation protocols and purification efficiency for obtaining specific cell types to generate precise disease models with cytopathic cell types. For disease modeling, single-cell RNA sequencing can help researchers identify and classify sporadic cases into sub-types for precise drug screening, as similarly performed for other diseases such as cancer [138]. The large-scale and systemic cross-comparison of sporadic iPSC-based neural disease phenotypes with whole genome sequencing (WGS) or whole exome sequencing (WES) could also help researchers address the correlations between specific disease phenotypes and genetic polymorphism. This combined strategy may be critical for the discovery of novel single nucleotide polymorphisms that are associated with the risk of developing neurodegenerative diseases.

### 8.9. Conclusions

Overall, current publications suggest that phenotypes could be recapitulated for most iPSC-based familial neurodegenerative diseases, as well as some sporadic diseases. Challenges such as the differentiation efficiency, maturation period, recapitulation of sporadic/late-onset disease phenotypes, construction of CNS-like tissues, and stable systems for large-scale drug screening remain to be resolved. Single-cell RNA sequencing may provide a novel tool for obtaining high-purity cytopathic specific cell lineages that overcome the limitations learned from human CNS developmental principles. Small compounds enhance the speed of neuronal maturation, but more efficient compounds are needed. For disease phenotype recapitulation, aging and environmental factors should be included in iPSC-based models. The present strategies include the genetic induction of cellular aging and environmental factor-like treatments. Disease sub-grouping is extremely important for drug screening, especially for neurodegenerative diseases. Without this process, cases with similar symptoms could be incorrectly categorized as the same disease, leading to unreliable results in drug discovery or clinical studies. Grouping sub-types into clusters may also provide insights to explain the failure of clinical drugs that may target some specific disease sub-types. To generate cell–cell circuits within different cell types, coculture systems, microfluid chips, and 3D organoids containing neurons, glial cells, and immune cells should be developed for drug screening. Among them, chip systems may have the best potential for drug screening to reduce cell/reagent use; they can be combined with automatic robotic systems for large-scale applications. Among current models, 3D organoids are most similar to human tissues, but further research is needed to improve the modeling stabilities. In addition, knowledge learned from patients and models should be updated for more reliable model design for screening clinically effective drugs.

Although there are several challenges to be solved, iPSC-based neurodegenerative disease models are used for drug screening. Novel compounds found from iPSC-based models are already applied to clinical trials. Recent work has demonstrated the potential of iPSC-based models for research and discovery focused on neurodegenerative disease.

## Figures and Tables

**Table 1:** List of publications that applied pluripotent stem cells for neurodegenerative disease modeling.

Disease	Gene Mutation	Phenotype	Cell Type	Potential Compound	Reference
DS	Trisomy 21	Aβ accumulation Aβ aggregates increased pTau and total Tau Tau redistribution	FB neuron	F127-Bdph	[59]
DS	Trisomy 21	Aβ accumulation Aβ aggregates increased pTau and total Tau Tau redistribution	FB neuron		[60]
DS	Trisomy 21	reduced synaptic activity dysregulated synapses	FB neuron		[61]
DS	Trisomy 21	toxicity to neurons fails to promote neuronal ion channel maturation and synapse formation	astroglia	minocycline	[71]
AD	*APP* SR	Aβ40 accumulation increased pTau activated GSK3B large early endosomes accumulation	FB neuron		[62]
AD	*APP* SR	Aβ accumulation increased Aβ42/40 ratio Aβ oligomer accumulation increased ROS increased apoptosis	FB neuron astrocyte	DHA	[63]
AD	*PSEN1*	Aβ accumulation increased Aβ42/Aβ40 ratio	FB neuron	Anti-Aβ cocktail (bromocriptine, cromolyn, topiramate)	[64]
AD	*APP*	increased APP Aβ accumulation increased total and pTau	FB neuron		[65]
AD	*APP* *PSEN1*	Aβ accumulation increased pTau	FB neuron		[66]
AD	*PSEN1*	increased Aβ42/40 ratio	FB neuron		[67]
AD	*PSEN*	Aβ42 accumulation	FB neuron		[68]
AD	*APP*	increased pPKA increased pTau	FB neurons		[70]
AD	*PSEN1*	Aβ accumulation altered cytokine release dysregulated calcium homeostasis increased oxidative stress reduced lactate secretion	astrocyte		[72]
PD	*PINK1* *LRRK2*	mitochondrial dysfunction	DA neuron	rapamycin coenzyme Q10 GW5074	[73]
PD	*SNCA*	increased α-SYN	DA neuron		[74]
PD	*PINK1* *Parkin*	dendrite degeneration decreased tyrosine hydroxylase enlarged mitochondria α-SYN inclusions	DA neuron		[75]
PD	*LRRK2* SR	α-SYN accumulation neuron degeneration neurons maturation defects autophagic dysregulation	DA neuron		[76]
PD	*LRRK2*	mitochondrial DNA damage	Neuron and DA neuron		[77]
PD	*LRRK2*	α-SYN accumulation autophagy dysregulation	astrocyte		[78]
ALS	SR	dysregulation of mitochondrial gene expression	MN		[79]
ALS	SR	TDP-43 aggregates	MN		[80]
ALS	*TDP43*	neurite degeneration increased TDP-43 TDP-43 aggregations MN death	MN	anacardic acid	[81]
ALS	*FUS*	FUS redistribution MN hypoexcitability axonal transport defects	MN	HDAC6 inhibitor (tubastatin A, ACY-738)	[82]
ALS	*FUS*	FUS redistribution formation of stress granules	MN		[83]
ALS	*SOD1*	increased oxidative stress mitochondrial dysfunction increased ER stress	MN		[84]
ALS	*VAPB*	reduced VAPB	MN		[85]
ALS	*TDP43*	increased TDP-43 TDP-43 redistribution induce MN death	astrocyte		[86]
ALS	*C9ORF72*	nucleocytoplasmic transport defects	MN		[87]
ALS	*FUS* *TDP-43* *SOD1* *SR*	protein aggregates/redistribution caspase activation LDH leakage neurite degeneration increased ROS mitochondrial dysfunction MN death	MN	ropinirole	[88]
ALS	SR	fewer muscle contractions MN degradation apoptosis increased in the muscle	NMJ	rapamycin bosutinib	[90]
ALS	*SOD1*	SOD1 aggregates neurofilament dysregulation	MN		[91]
ALS	*SOD1* *TDP43 C9ORF72* SR	MN death autophagy dysregulation	MN	Src/c-Abl pathway inhibitor (bosutinib)	[92]
ALS	*SOD1*	induce MN death	oligodendrocyte		[93]
ALS	*SOD1* OE	SOD1 inclusion neurite degeneration astrocyte induced MN death	MN astrocyte		[94]
ALS	*SOD1*	disrupted EphB1–ephrin-B1 pathway	astrocyte		[95]
HD	*HTT*	decreased cadherin, TGF-β, BDNF caspase activation	FB neuron		[98]
HD	*HTT*	electrophysiology change metabolism dysregulation decreased cell adhesion neuron death	NSC GABA neuron		[99]
HD	*HTT*	proteasome inhibition	GABA neuron		[100]
HD	*HTT*	HTT aggregates increased lysosomes/autophagosomes increased nuclear indentations neuron death	GABA neuron		[101]
SCA6	*CACNA1A*	increased Cav2.1 decreased α1ACT TH depletion induced vulnerability	Purkinje cell	TRH Riluzole	[102]
SCA3	*ATXN3*	ATXN3 aggregates	non-Purkinje neuron		[103]
SCA3	*ATXN3*	autophagy dysregulation	non-Purkinje neuron		[104]
SMA	*SMN*	decreased SMN decreased neurite decreased synaptic maturation	neuron	VPA and tobramycin	[105]
SMA	*SMN*	decreased UBA1 UBA1 redistribution decreased MN differentiation.	MN		[106]
SMA	*SMN*	decreased SMN neurite degeneration excitability dysfunction	MN		[107]
SMA	*SMN*	impaired AChR	NMJ	VPA and PMOs	[108]
SMA	*SMN*	abnormal calcium regulation reduced neurotrophic factors	astrocyte		[109]
AxD	*GFAP*	Rosenthal fiber-like structures increased inflammatory cytokine release	astrocyte		[110]
AxD	*GFAP*	GFAP aggregates inhibit proliferation of oligodendrocyte progenitor cells reduce oligodendrocyte progenitor cells myelination potential	astrocyte		[111]

AChR: acetylcholine receptor; AD: Alzheimer’s disease; AxD: Alexander disease; ALS: amyotrophic lateral sclerosis; APP: amyloid precursor protein; ATXN3: ataxin 3; Aβ: amyloid beta; α1ACT: C-terminal of Cav2.1; α-SYN: α-synuclein; BDNF: brain-derived neurotrophic factor; Bdph: *n*-butylidenephthalide; Cav2.1: Cav2.1 P/Q voltage-dependent calcium channel; DA neuron: dopaminergic neuron; DHA: docosahexaenoic acid; DS: Down syndrome; ER: endoplasmic reticulum; FB: forebrain; FUS: RNA-binding protein FUS/TLS; GFAP: glial fibrillary acidic protein; HD: Huntington’s disease; HDAC6: histone deacetylase 6; HTT: huntingtin; LDH: lactic dehydrogenase; MN: motor neuron; NSC: neural stem cell; NMJ: neuromuscular junction; OE: overexpression; PD: Parkinson’s disease; PKA: protein kinase A; PMOs: phosphorodiamidate morpholino oligonucleotides; p-Tau: phosphorylated Tau protein; ROS: reactive oxygen species; SCA: spinocerebellar ataxia; SMA: spinal muscular atrophy; SMN: survival motor neuron protein; SOD1: superoxide dismutase 1; SR: sporadic; TDP-43: transactive response DNA binding protein 43; TGF-β: transforming growth factor beta; TH: thyroid hormone; TRH: thyrotropin releasing hormone; UBA1: ubiquitin-like modifier activating enzyme 1; VAPB: vesicle-associated membrane protein-associated protein B/C; VPA: valproic acid.
